# Determinants of Plasma Docosahexaenoic Acid Levels and Their Relationship to Neurological and Cognitive Functions in PKU Patients: A Double Blind Randomized Supplementation Study

**DOI:** 10.3390/nu10121944

**Published:** 2018-12-07

**Authors:** Hans Demmelmair, Anita MacDonald, Urania Kotzaeridou, Peter Burgard, Domingo Gonzalez-Lamuno, Elvira Verduci, Melike Ersoy, Gulden Gokcay, Behiye Alyanak, Eva Reischl, Wolfgang Müller-Felber, Fabienne Lara Faber, Uschi Handel, Sabrina Paci, Berthold Koletzko

**Affiliations:** 1 Division Metabolic and Nutritional Medicine, LMU-Ludwig-Maximilians-Universität Munich, Dr. von Hauner Children’s Hospital, 80337 Munich, Germany; Wolfgang.Mueller-Felber@med.uni-muenchen.de (W.M.-F.); Fabienne.Faber@med.uni-muenchen.de (F.L.F.); Uschi.Handel@med.uni-muenchen.de (U.H.); 2 The Children’s Hospital Birmingham, Birmingham B4 6NH, UK; Anita.Macdonald@nhs.net; 3 Division of Neuropediatrics and Metabolic Medicine, Heidelberg University Hospital, 69120 Heidelberg, Germany; Urania.Kotzaeridou@med.uni-heidelberg.de (U.K.); Peter.Burgard@med.uni-heidelberg.de (P.B.); 4 Department of Pediatrics, IDIVAL-Hospital M. Valdecilla, 39008 Santander, Spain; domingo.gonzalez-lamuno@unican.es; 5 Department of Pediatrics, San Paolo Hospital Milano, 20142 Milano, Italy; elvira.verduci@unimi.it (E.V.); sabrina.paci@asst-santipaolocarlo.it (S.P.); 6 Department of Pediatric Nutrition and Metabolism, Istanbul Medical Faculty, Istanbul University, 34093 Istanbul, Turkey; zeynepcey@hotmail.com (M.E.); guldengokcay@gmail.com (G.G.); 7 Department of Child Psychiatry, Istanbul Medical Faculty, Istanbul University, 34093 Istanbul, Turkey; behiyealyanak@hotmail.com; 8 Research Unit of Molecular Epidemiology, Institute of Epidemiology, Helmholtz Zentrum München, 85764 Neuherberg, Germany; eva.reischl@helmholtz-muenchen.de

**Keywords:** phenylketonuria, docosahexaenoic acid, cognitive function, motor skills, neurological function

## Abstract

Children with phenylketonuria (PKU) follow a protein restricted diet with negligible amounts of docosahexaenoic acid (DHA). Low DHA intakes might explain subtle neurological deficits in PKU. We studied whether a DHA supply modified plasma DHA and neurological and intellectual functioning in PKU. In a double-blind multicentric trial, 109 PKU patients were randomized to DHA doses from 0 to 7 mg/kg&day for six months. Before and after supplementation, we determined plasma fatty acid concentrations, latencies of visually evoked potentials, fine and gross motor behavior, and IQ. Fatty acid desaturase genotypes were also determined. DHA supplementation increased plasma glycerophospholipid DHA proportional to dose by 0.4% DHA per 1 mg intake/kg bodyweight. Functional outcomes were not associated with DHA status before and after intervention and remained unchanged by supplementation. Genotypes were associated with plasma arachidonic acid levels and, if considered together with the levels of the precursor alpha-linolenic acid, also with DHA. Functional outcomes and supplementation effects were not significantly associated with genotype. DHA intakes up to 7 mg/kg did not improve neurological functions in PKU children. Nervous tissues may be less prone to low DHA levels after infancy, or higher doses might be required to impact neurological functions. In situations of minimal dietary DHA, endogenous synthesis of DHA from alpha-linolenic acid could relevantly contribute to DHA status.

## 1. Introduction

Children with classical phenylketonuria (PKU, OMIM 261600) diagnosed by newborn screening who receive adequate dietary treatment from the neonatal age onwards generally reach the normal range of neurological and cognitive functioning, even though some differences in performance when compared to healthy siblings have been demonstrated [1,2]. The PKU diet is based on a strictly limited intake of natural protein, supplemented with a phenylalanine (Phe) free amino acid mixture. The PKU diet generally excludes protein rich animal foods, such as dairy products, meat, eggs, and fish, which are natural sources of the long chain polyunsaturated fatty acids (LC-PUFA) [3]. Humans can endogenously produce LC-PUFA from precursor essential fatty acids, but the rate of conversion is limited and dietary intake is the key determinant of blood levels of the *n*-3 long-chain polyunsaturated fatty acid docosahexaenoic acid (DHA). Children with PKU often have significantly lower blood levels of DHA than healthy controls [4,5,6,7].

Many studies in infancy have demonstrated the importance of an adequate LC-PUFA status for optimal neurological development, even though such effects have not been confirmed in all studies [8]. LC-PUFA seem most important for neurological functions early in life and in the elderly, while a functional benefit of LC-PUFA for children has remained controversial [9,10]. Supplementation studies have focused on DHA, which is found in high amounts in the brain and the eye, where DHA is important for membrane function, interacts with PPARs, is associated with neurotransmitter levels, and acts as a precursor for docosanoids [11,12]. The variable results observed from studies investigating the effect of DHA supplementation in children on cognitive or neurological functioning may in part be due to confounding baseline DHA intake and status, as well as genetic variation in the activity of the fatty acid desaturases (FADS) and elongases involved in LC-PUFA synthesis [9]. The low baseline levels in PKU children suggest that they might benefit more from DHA than healthy children. In several studies, neurological and cognitive improvements have been observed after supplementing children with PKU with *n*-3 LC-PUFA [13,14,15]. Visually evoked potential latencies have been improved with fish oil supplementation or a blend of *n*-3 and *n*-6 LC-PUFA [14,15]. A common feature of these studies is a high dose of DHA (≥10 mg/kg&day) and the application of mixtures of eicosapentaenoic acid (EPA) and DHA. The question about the DHA dosage required to achieve a beneficial effect has remained unanswered.

The very low LC-PUFA intake in non-supplemented patients with PKU provides the opportunity to study the relationship between plasma levels of essential fatty acids and their desaturation and elongation products in the almost complete absence of the influence of dietary LC-PUFA. It has been established that different genetic variants of ∆6-desaturase and ∆5-desaturase influence LC-PUFA levels, with up to 30% of the variance of blood arachidonic acid (ARA) explained by single nucleotide polymorphisms (SNPs) in the FADS gene cluster that predict the activity of desaturating enzymes [16,17]. In addition to SNPs in the FADS1/FADS2/FADS3 gene cluster on chromosome 11, SNPs of genes encoding fatty acid elongases also influence LC-PUFA levels [17]. 

As the same enzymes catalyze the conversion of *n*-6 linoleic acid (LA) to ARA and of *n*-3 α-linolenic acid (ALA) to DHA, it may be expected that there is a similar importance of the FADS genotype for the corresponding fatty acid levels. However, there is a much smaller influence of the FADS genotypes on DHA than on ARA levels. An observational study explained only 6% of the variation of red blood cell DHA content in pregnant women by the FADS genotype [18], while other studies found no significant influence of FADS genotypes on DHA levels [19,20]. This agrees with the well-known importance of the dietary intake of DHA and EPA for blood levels [21].

We aimed to assess the effects of different dosages of DHA supply in PKU children on fatty acid levels in blood lipids and on neurocognitive outcomes.

## 2. Materials and Methods 

The study was conducted as a double blind randomized multicentric clinical trial with an intervention period of six months. Randomization was stratified for each study center. Patients with established PKU were enrolled in six major metabolic centers treating PKU children in Germany (Heidelberg, Munich), Italy (Milan), the UK (Birmingham), Turkey (Istanbul), and Spain (Santander). PKU patients attending the corresponding outpatient departments and considered eligible were invited to participate. All parents consented in writing to participate after detailed verbal and written information was given. The study was conducted in accordance with the Declaration of Helsinki, and the protocol was approved by the local Ethics committees of all participating hospitals and was registered with clinical trials.gov (NCT00909012).

### 2.1. Inclusion and Exclusion Criteria

Eligibility criteria included patients aged from 5 to 13 years, a diagnosis of classical PKU, continuous treatment from the newborn period, good metabolic control demonstrated by dietary adherence with a low Phe diet and at least two plasma Phe measurements with an average below 480 µmol/L during the six-month period prior to study enrollment, weight between the 3rd and 97th percentile according to the WHO standard, and a natural protein tolerance of ≤12 g/day. Patients with additional co-morbidities or who consumed *n*-3 LC-PUFA supplements (separately or added to L-amino acid supplement) were excluded from participation.

### 2.2. Intervention

The dietary intervention consisted of the randomized allocation to one of five types of capsules providing 0, 20, 43, 80, or 127 mg of DHA per capsule in the form of triacylglycerols. Capsules were prepared by Nutricia (Liverpool, UK) by mixing appropriate amounts of high oleic sunflower oil and algal oil. The algal oil contained only trace amounts of LC-PUFA other than DHA, with all concentrations below 0.1% (Supplementary Table S1). With the LA percentage in the supplements ranging from 3.5% to 5.8%, the additional LA intake via formula for all children was below 60 mg per day. Subjects with a body weight below 35 kg consumed one capsule daily and participants weighing 35 or more kg consumed two capsules daily. The different DHA contents per capsule and the different numbers of capsules per day aimed to achieve a daily DHA intake from 0 to 7 mg per kg body weight. Allocation concealment was achieved by the addition of orange flavor to all capsules.

### 2.3. Outcome Assessments

Before the start of the capsule intervention, baseline examinations were performed and venous blood was collected. As an indicator of neurological function, latencies of visually evoked potentials (VEP) were recorded using a suitable device for recording pattern reversal, with a filter setting of 1 to 100 Hz, sensitivity of 20 μV/div, and time base of 30 ms/div. The patients were exposed to alternating black and white checkerboard patterns, with its size modified from 120′ to 7.5′. All recordings were performed twice, with 50 stimulations each. Sweeps were continued until the averaged potential stabilized. Available equipment varied between study centers, so applied procedures could not be strictly standardized, but a pattern size of 15′ was used in all centers. Electrograms were evaluated and latencies and amplitudes of the P100 wave were recorded by two experienced neurologists. Only if both investigators rated the electrogram as acceptable were data included in the database. As outcome parameter P100, latency (ms) of the 15′ pattern size was defined a priori.

Raven’s Progressive Matrices (RPM) were used to assess cognitive performance. Depending on the local availability for the corresponding age range, standard progressive matrices or colored progressive matrices were applied [22]. Although not all children could be tested with identical procedures, care was taken to apply the same test pre and post intervention for each child and to ensure a similar test environment. While this precluded a comparison between the centers, this procedure seemed suitable for evaluating changes over time. 

The fine and gross motor skills were tested with the Lincoln-Oseretzky Motor Development Scale (LOS-SF 18). The LOS corresponds to the Motometric Rostock-Oseretzky Scale by Kurth, as described in detail by Beblo et al. [13], but provides the advantage of commercially available standardized test equipment. The sum of raw scores from 18 subtests (e.g., coin sorting, clapping, and walking on a thin line) is converted to age-related standardized T-Scores (mean 50, SD = 10). The same investigator examined the child at baseline and post-intervention in the same room. Parents could only attend the investigation to support their children. Investigators from all centers participated in a videotaped training session. 

Medical treatments, physical examinations, and anthropometric measurements were determined by the individual study centres. Caregiver reports of the children’s competencies and behavior problems were obtained via the Child Behavior Checklist/ 4-18 (CBCL) [23].

### 2.4. Biochemical Measures

Non fasted venous blood samples were collected in plain and EDTA coated tubes and serum and plasma were obtained according to study center routines. From the serum samples, cholesterol, LDL cholesterol, HDL cholesterol, triacylglycerol, and Phe levels were determined at the centers by the local clinical chemistry units. From the EDTA, blood plasma aliquots were used for the determination of glycerophospholipid (GPL) fatty acids. The cell sediment was used for extracting DNA for genotyping from the leucocytes. For these analyses, plasma and cell sediment samples were sent on dry ice to the Dr. von Hauner Children’s Hospital, Munich and the Helmholtz-Zentrum, Munich, respectively.

Quantification of GPL fatty acids in plasma was performed as described before [24]. Selective preparation of methyl ester derivatives of glycerophospholipid bound fatty acids was achieved by co-precipitation of triacylglycerols and cholesterol esters with proteins and base catalysed transesterification, excluding methyl ester synthesis from non-esterified fatty acids. Gas chromatographic analysis using a 50 m long BPX70 column with a 0.22 mm inner diameter (SGE Europe, Milton Keynes, UK) enabled the quantification of 29 fatty acids with a chain length from 14 to 22 carbon atoms, including 14 saturated or monounsaturated fatty acids; the polyunsaturated fatty acids of the *n*-6 series: C18:2cc, C18:2tt, C18:3, C20:2, C20:3, C20:4, C22:4, and C22:5; from the *n*-3 series: C18:3, C18:4, C20:3, C20:5, C22:5, and C22:6; and the *n*-9 polyunsaturated C20:3. The coefficients of variation observed for the analysis varied from 1.8% to 30%, and for DHA, a coefficient of variation of 6.2% was obtained (*n* = 11).

### 2.5. Genotyping

The extraction of genomic DNA from the cell sediment was performed by a standard salt-precipitation method. Genotyping was conducted using matrix-assisted laser desorption/ionization time-of-flight mass spectrometry (MALDI-TOF MS) to detect allele-specific primer extension products (Mass Array, Agena Bioscience, San Diego, CA, USA). Validated standard protocols according to Agena Bioscience’s instructions were applied.

SNPs for genotyping in the FADS gene cluster and fatty acid elongase 5 were selected on the basis of positional and functional aspects, as described before [16]. Genotyping data were analyzed with Sequenom’s Typer Analyzer Software package.

### 2.6. Sample Size Estimation and Data Analysis

The number of patients recruited was planned to test a beneficial effect of at least 4 mg/kg&day DHA intake by a paired comparison of VEP latencies pre and post intervention. Assuming an intra-individual correlation of VEP latencies of 0.5 and considering the standard deviation of P100 latencies of 6.2 ms, as observed by Beblo et al. [14], a clinically relevant change of 4 ms could be expected to be detected as statistically significant with a probability of 80.3% (α = 0.05, two tailed test) from repeated measurement of a sample of 21 subjects. 

Thus, already with 21 subjects, the study would have been adequately powered to detect an effect of a 4 mg/kg&day DHA intake, but as we aimed to identify a dose-response relationship between DHA intake and the functional outcome VEP latency, we had to recruit more subjects and allocate them to different doses of DHA intake to cover the range of intakes from 0 to 7 mg/kg&day for the detection of potential dose-response relationships. We estimated that with 125 subjects with different intakes per kg, including at least 21 subjects in a placebo group, we would be able to identify a linear association between DHA intake and VEP latency. 

Data were recorded on forms for later incorporation into an electronic database (Microsoft Excel). In the database, the results of the laboratory analyses and the outcome of the neurological and cognitive testing were also added. After the entry of all data and accuracy checking, the database was closed. Thereafter, the dose allocation was unblinded.

### 2.7. Statistical Analyses

Results are presented as mean ± standard deviation. For comparisons between subgroups of subjects, ANOVA or chi^2^-tests have been applied for continuous or categorical variables, respectively. Correlations according to Pearson were used to describe associations. The influence of DHA intake on the outcome parameters was tested by multiple linear regression analysis using the pre-study values and the DHA intake as predictors and by paired t-tests comparing pre and post values stratified according to DHA intake. Factors such as age, plasma Phe concentration, and genetic polymorphisms, were tested by linear regression analysis for an influence on the outcome parameters. *P*-values of less than 0.01 were considered as statistically significant, where appropriate *p*-values were adjusted for multiple testing according to Bonferroni.

Statistical calculations were performed using the Software Package for Social Sciences (Version 22, SPSS Inc. Chicago, IL, USA) and for testing for Hardy-Weinberg equilibrium and the calculation of allele frequency, the Chi-sq Hardy-Weinberg equilibrium test calculator available online for biallelic markers was applied [25].

## 3. Results

**Figure 1:**
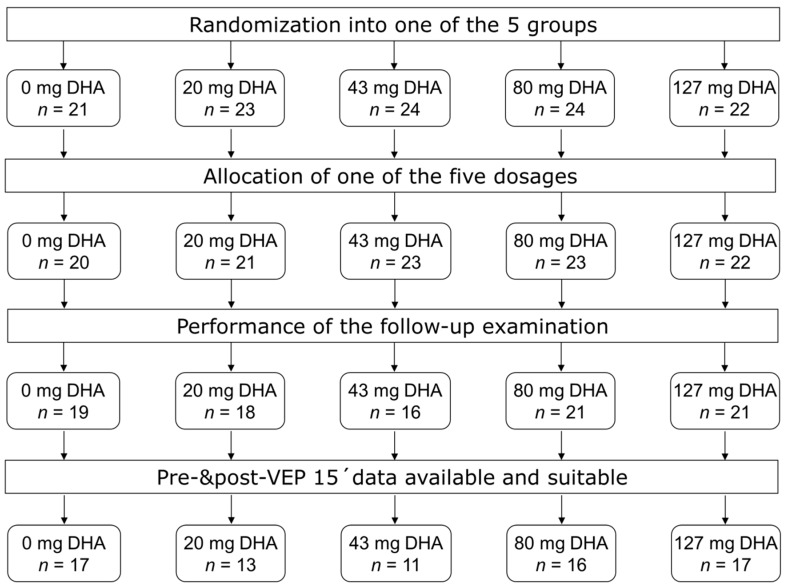
Participant flow chart according to CONSORT.

From Jan 2008 to July 2010, 114 subjects from six study centers were enrolled in the study (Figure 1). After the pre study examination and biosample collection, 109 began the intervention (Figure 1). Post-intervention assessments of VEPs and tests of cognitive function and motor skills were completed by 95 children (Figure 1). Fourteen children terminated participation because parents withdrew consent (*n* = 7), were lost to follow up (*n* = 3), or for unspecified reasons (*n* = 4). Some of the performed tests did not meet the preset quality control criteria and were excluded from the evaluation, which left 74 PKU children with a complete VEP15 dataset, 78 LOS datasets, and fully evaluable RPM data in 80 children. Fatty acid analyses including pre and post intervention data could be evaluated from 82 children. The evaluated children from the individual study centers are described in Table 1. There were significant differences between the study centers with respect to fatty acid status and baseline values of outcome tests, but analysis of variance did not reveal a significant effect of study centers on the change of the parameters during the study (Supplementary Table S2). 

The percentage of subjects with usable data for the primary outcome differed significantly between study centres (*p* = 0.003, chi^2^-test), from 44% to 93%. Mean parentally reported numbers of consumed capsules as an indicator of compliance with the study protocol ranged from 96% (Birmingham, Santander) to 102% (Istanbul), with a mean of 98% of the number of capsules to be consumed, without significant differences between the study centres (*p* = 0.08, ANOVA). No severe adverse side effects and no vomiting due to capsule intake were reported. Three children reported nausea and six children experienced fishy regurgitation. Occurrence of adverse events was not associated with higher DHA intake.

**Figure 2:**
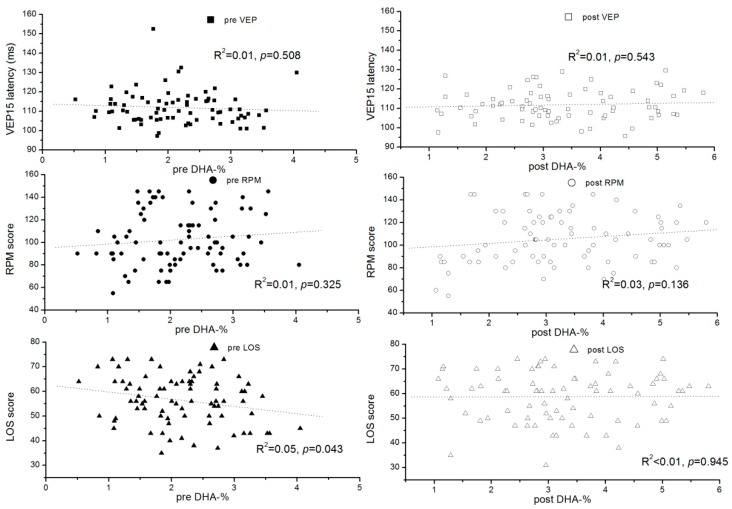
Association of the latency of visually evoked potentials with pattern size 15′ (VEP15 latency), the Ravens score (RPM), and the Lincoln-Oseretzky score (LOS) with the percentage of DHA in plasma glycerophospholipids before (pre) and after (post) intervention.

**Figure 3:**
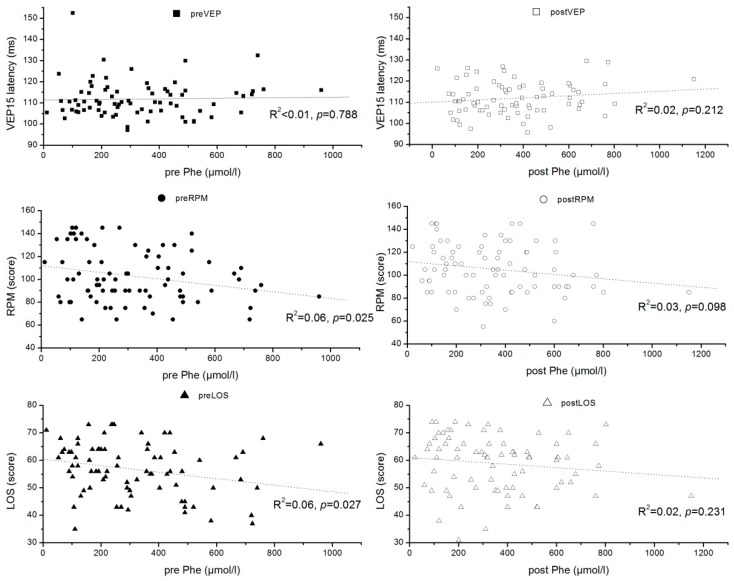
Association of the latency of visually evoked potentials with pattern size 15′ (VEP15 latency), the Ravens score (RPM), and the Lincoln-Oseretzky score (LOS) with the plasma phenylalanine (Phe) concentrations (µmol/L) before (pre) and after (post) intervention.

The effect of DHA dose on the outcome parameters was studied using multiple linear regression considering the pre-values and DHA dosage per kg of body weight (Table 2). While functional outcomes and fatty acid status after intervention were all strongly associated with the corresponding pre study results, only DHA concentration and percentage values were significantly associated with DHA intake. However, there were no significant relationships between DHA intake and functional outcome parameters, although between 33% (plasma TG) and 64% (RPM) of the post intervention variances were explained by the model (Table 2). The latency at pattern size 15′, latencies at other pattern sizes, and amplitudes of the VEPs were not influenced by the intervention (Supplementary Table S3). The influence of the DHA status on the neurological and cognitive outcomes was further investigated by relating DHA levels at baseline and after intervention to these parameters, which did not identify any significant relationship (Figure 2). Pre and post intervention VEP 15 latencies were unrelated to Phe levels, but LOS and RPM scores showed a tendency to decrease with increasing Phe levels (Figure 3). Nevertheless, in univariate analyses, maximally 6% of the variance in an outcome parameter (pre value of RPM) was explained by the Phe levels.

A potential influence of DHA intake was also investigated by comparing changes after stratification into supplementation groups with 0 mg DHA/kg&day, >0 mg DHA/kg&day but <1.9 mg/kg&day, and ≥1.9 mg/kg&day (Table 3). The stratification into groups with no, lower, and higher intakes did not show significant group differences between the changes, but a trend towards a higher RPM increase in the zero-intake group. Only in the group with zero DHA intake (placebo group) was the change of the LOS T-score significantly different from 0, while all other changes were only minor and not significantly different from 0 (Table 3). 

A total of 22 SNPs previously related to ∆6 desaturation, ∆5 desaturation, or fatty acid acyl chain elongation were considered in respect to their influence on ARA, DHA, VEP15 latency, RPM score, and LOS T-score. After correction for multiple testing, only ARA levels were significantly influenced by some SNPs based on linear regression analyses without considering potential confounding factors (Table 4). Considering adjustment for multiple testing only for ARA levels, significant associations with genotypes could be found and for none of the tested SNPs was a significant association with DHA or functional outcomes found. 

**Figure 4:**
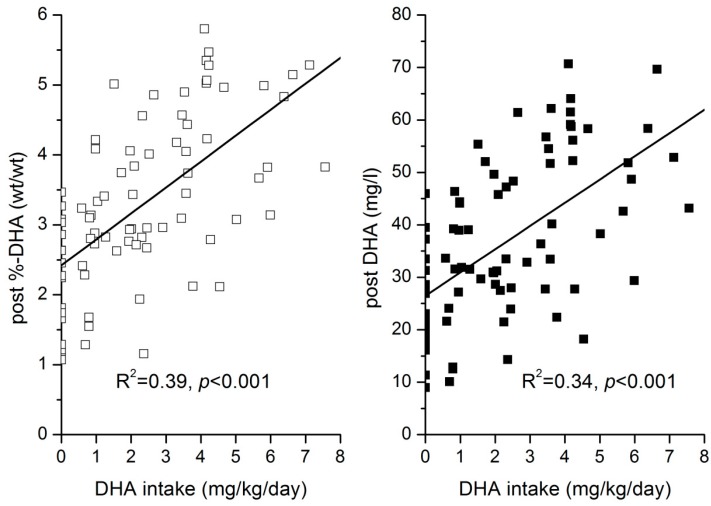
Linear relationship between DHA intake (mg/kg&day) and post intervention phosphoglycerid bound DHA concentration (□) and percentage contribution of DHA to total analysed glycerophospholipid fatty acids (■).

**Figure 5:**
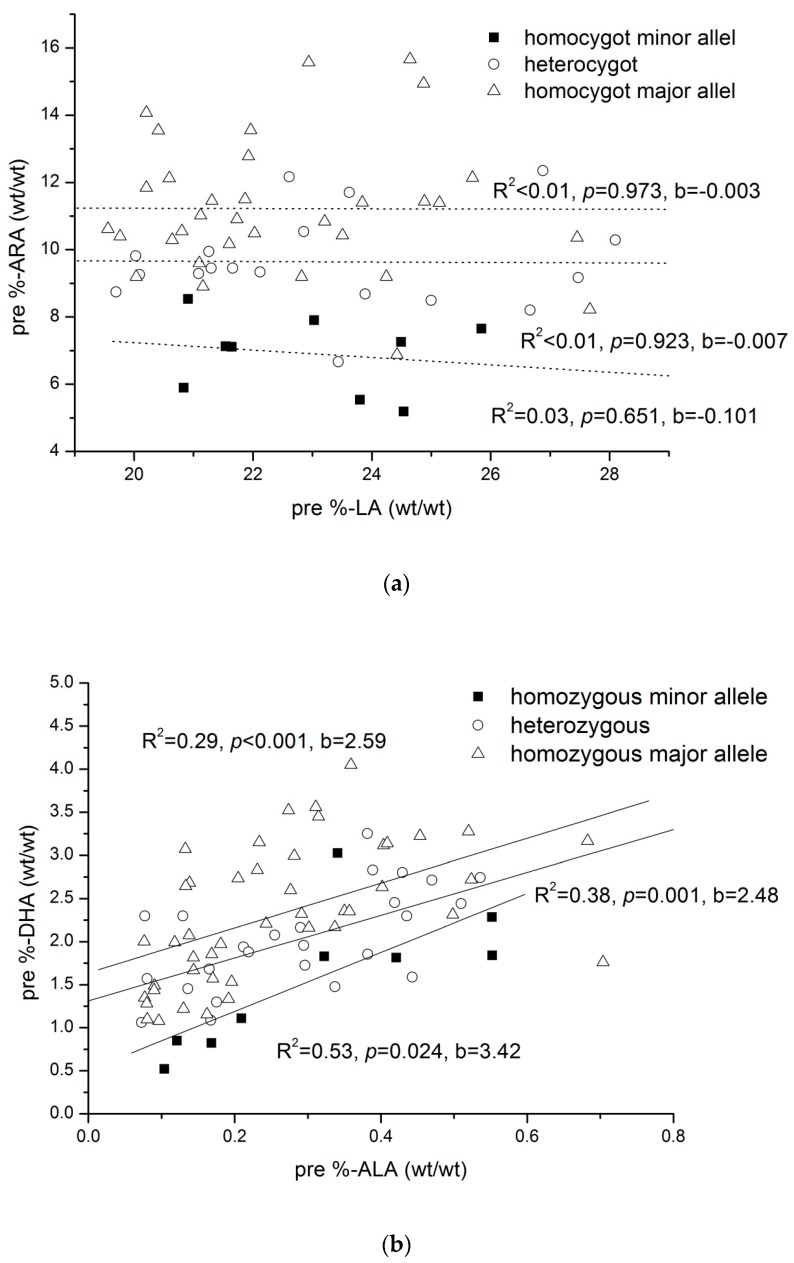
(**a**) Pre study arachidonic acid (%-ARA) percentages versus linoleic acid (%-LA) percentages in glycerophospholipids according to the individual alleles of rs174548; (**b**) Pre study docosahexaenoic acid (%-DHA) percentages versus α-linolenic acid (%-ALA) percentages in glycerophospholipids according to the individual alleles of rs174548.

DHA intake was linearly related to absolute DHA concentrations and DHA-% in plasma GPLs over the whole tested dose range (Figure 4). Multiple regression analysis was used to investigate the influence of the rs174548 genotype, corresponding essential precursor fatty acid concentrations, total concentration of determined GPL bound saturated fatty acids, and DHA intake on the major *n*-3 and *n*-6 LC-PUFA. Saturated fatty acids, but not monounsaturated fatty acids, were included in the model to account for the possibility that LC-PUFA incorporation into GPLs could be influenced by the availability of saturated fatty acids to be combined with essential fatty acids or LC-PUFA in plasma GPL. The models were set up for the pre- and post-intervention data (Table 5). For all considered fatty acids, significant models were identified. The variance explained by these factors ranged from 36% for *n*-6 docosapentaenoic acid (DPA) to 84% for EPA in the pre-intervention data and from 43% for ARA to 90% for EPA in the post-intervention data. Post-intervention DHA intake was significantly positively related to DHA levels and significantly negatively related to ARA and *n*-6 DPA. Post-intervention, the different DHA intakes during the study period (0 mg/kg&day to 7 mg/kg&day) were the major determinants of DHA levels. ALA levels showed the highest β in the pre-intervention situation and were found to be related to DHA concentration. The Rs174548 genotype was related to most LC-PUFA concentrations, and significant interactions between genotype and essential precursor fatty acid concentrations were seen for EPA, *n*-3 DPA, DGLA (pre only), and DHA (pre only). Interestingly, ARA was only related to the genotype and not to the LA concentration (Figure 5a), while in the case of DHA, the ALA precursor level and genotype were both significantly related to DHA (Figure 5b). 

## 4. Discussion

Dietary intake of DHA led to a proportional increase of DHA in plasma GPLs, but a beneficial effect of improved DHA status on the tested cognitive and neurological functions in children with PKU was not seen with the dosage range tested. The study confirmed the high importance of the FADS genotype for the LC-PUFA status. It also demonstrated that in the absence of an exogenous supply of LC-PUFA, the availability of the precursor fatty acid ALA modulates DHA status. 

The lack of significant improvement of neurological and cognitive function in the PKU children is in line with observations in healthy children and adolescents, which often do not report a significant benefit of DHA or fish oil supplementation [26]. Our intervention can be compared with *n*-3 supplementations in healthy children, which have typically applied dosages from 100 to 1200 mg per day [27,28,29,30,31,32] and even higher doses in studies with patients suffering from various disorders [33]. This corresponds to dosages of around 20 mg/kg; thus, our applied dosage of maximally 7.5 mg/kg was comparatively low, even though we used pure DHA and not a mixture of DHA and EPA. As the majority of the participating children received DHA dosages clearly below 250 mg per day, our findings are in line with the EFSA scientific opinion that the dietary DHA intake of children from 2 to 18 years should be 250 mg [34]. Thus, based on our data, an optimal intake cannot be defined.

Beblo et al. reported, after supplementing PKU children for three months with more than 15 mg/kg&day, DHA percentages of DHA in plasma phospholipids of 7.1 ± 0.1% (M ± SEM), while we saw maximal values of around 5.5% [13]. The initial DHA percentages in our study participants showed considerable differences between the study centres, with very low values in some centres, whereas in Munich, Heidelberg, and Birmingham, DHA values were only slightly lower than the mean 2.9% DHA found in healthy omnivorous German children [35]. In contrast, average ARA values for all study centres were higher than those observed in healthy children, confirming previous observations that PKU children present with low DHA, but with normal or high ARA values [4,36]. The essential fatty acid LA was similar to the healthy children in most study centres, but in Milan, values were low and Istanbul, they were high (25%). For ALA, there were two different trends among study centres, with mean values <0.2% in Istanbul, Santander, and Milan, and mean values >0.3% in both German centres and in Birmingham. The German cohort of healthy children had 0.22% (0.10) [median (IQR)] ALA in their GPL fatty acids [35], which indicates that ALA supply was good in some centres, but low in others, presumably reflecting the use of different vegetable oils in the diet. Our data confirm previous findings that PKU patients show lower *n*-3 LC-PUFA than healthy controls, but the differences between healthy omnivorous children and the PKU patients were small in some centres. The DHA data from Istanbul, Santander, and Milan confirm the findings of the 2013 Lohner review [7], while German and UK sites support recent observations from a German PKU study that there was no significant difference between patients and controls [37]. 

Animal studies and several studies in humans indicate beneficial effects of DHA on measures of cognitive and neurological functions [38], and this might also apply to PKU patients, who very early on, develop low DHA levels without an intake of DHA [39]. As no fully standardised reference method was applied and as we did not include a healthy reference group, we cannot demonstrate that the observed latencies of the VEPs were longer than in healthy controls, but this is suggested by a comparison with the findings of Beblo and Agostoni, who found similar latencies in PKU patients which differed from healthy controls [14,15]. For the IQ testing, there is a broad variance of the results, but we did not observe mean test results below the expected norm. The LOS test for motor skills yielded results which tended to be above average, providing no indications of deficits in PKU children [40], as reported previously [13]. The CBCL scores did not give reasons to exclude a participant, but otherwise, we did not further consider this parameter in the evaluations. As for other parameters, there were clear differences between the centres. Additionally, the study participants had no clinically relevant abnormalities of triglyceride and cholesterol levels, which did not significantly change during the intervention period.

The statistical evaluation did not reveal significant improvements of any of the tested neurological and cognitive functions, but rather a considerable stability of the test results. While DHA intake was not significantly associated with post intervention LOS T-scores, VEP latencies, or IQ, between 40% and 64% of the post intervention variance was explained by the pre value. In an alternative evaluation, a comparison of the change of the neurological outcomes of the children with DHA intakes of at least 1.9 mg/kg to those without DHA intake did not indicate any significantly greater improvement in supplemented children. Thus, the data indicate reproducible and valid measurements, but no systematic benefit from the DHA supplementation. 

It was previously shown that infants with the FADS genotype associated with less endogenous production of LC-PUFA particularly benefit from the provision of exogenous DHA [41], so we took genotype into account in our evaluations, but this did not change the findings. Similarly, analyses stratified according to the study centres (data not shown) did not indicate an effect of DHA supplementation. A further indication that in the range studied, DHA status and intake do not influence neurological and cognitive outcomes, is that neither basal nor post intervention significant associations between GPL DHA and the outcomes could be found. According to observations in non-human primates [42], six months of supplementation should allow for brain DHA to adapt to supplementation and leave time to improve cognitive function. 

In agreement with the observations in the general population, DHA levels were clearly affected by DHA supply [43]. The observed proportionality of the dosage of DHA supply with the percentage increase in blood lipids confirms observations in adults and pregnant women given higher doses of DHA+EPA [44,45]. Considering the influence of body weight on the DHA concentration changes observed after supplementation [46] and the range of body weights in children aged 5 to 13 years, we related DHA change to DHA intake per kg body weight and found a linear relationship over the full tested range up to 7 mg/kg. This is in agreement with an observation in adults who consumed fish oil (EPA/DHA = 6/4) for five months and showed a linear association between intake and red blood cell omega-3 index up to a daily intake of 1800 mg, corresponding to 30 mg/kg [47].

In adult studies, the increase of the DHA status as % or concentration was not only dependent on the intake, but also on the baseline level, with a negative association between baseline and increase [44,47]. In our data, baseline DHA and daily intake had a similar influence on the post-supplementation DHA and together, these factors explained about two thirds of plasma GPL DHA variance. Although the change of DHA was inversely associated with the baseline level, the pre-value and the dose were both positively associated with the post-value. Thus, we can assume that there are two different sources of DHA which define the post-value, i.e., the study related supplement intake and the endogenous synthesis. It has been shown that FADS expression in the liver is attenuated by DHA [48], thus more reduction of endogenous synthesis can be expected from participants with high initial DHA levels driven by endogenous synthesis, as observed here. Furthermore, in the absence of dietary DHA, as given at the baseline, it can be expected that the endogenous DHA production is highest, indicating that the DHA levels are achievable without exogenous supply.

Previous studies in humans with stable isotopes have found variable, but generally low, conversion rates of ALA to DHA [49,50], which seem insufficient to provide an optimal brain DHA content. This is supported by the finding that ALA supplementation does not lead to a significant increase of DHA levels in most studies [49]. Since DHA levels are associated with fish or supplement intake [51], it is evident that optimal DHA levels depend on DHA intake [52]. Nevertheless, recent studies in mice have suggested that endogenous synthesis from ALA provided by a 2% ALA (weight-% of dietary fat) diet can only establish an 8% lower brain DHA content compared to a diet containing 1.8% preformed DHA [53]. 

Nevertheless, to some degree, low LA combined with high ALA intake can increase DHA levels in humans [54]. A constant infusion of non-esterified labelled ALA showed that the conversion rate of ALA to DHA was the same in mice fed 3% ALA of all dietary fatty acids as in mice fed a diet with only 0.1% ALA. Interestingly, in mice fed a 10% ALA diet, the conversion rate decreased and DHA levels were the same as with the 3% diet [55]. Endogenous DHA synthesis is considered to be lower in humans than in mice [56], but it seems to contribute to DHA status. Although the efficacy of the direct intake of preformed DHA is much stronger, endogenous ALA conversion seems to be relevant, particularly if the DHA intake is very low, as in non-supplemented PKU patients. The importance of ALA as a source for DHA was reported for PKU patients who showed a significant increase of their DHA levels after an increase of the ALA intake relative to the LA intake [57].

In our study, the multiple regression analyses indicated that DHA levels were significantly associated with ALA levels in the absence of DHA intake, and also with a DHA intake up to 7 mg/kg body weight per day. A relevant endogenous DHA synthesis is also indicated by the influence of the FADS genotype on DHA levels, if considered jointly with the precursor level. For *n*-6 fatty acids, the level of the precursor LA is barely related to the level of the conversion product ARA, while genotype is of significant importance [19,58]. This indicates that it is not the precursor, but the enzymatic conversion capacity, which primarily limits ARA levels, which contrasts to the *n*-3 side. Interestingly. in our model, which took the already previously observed correlation among all phospholipid bound fatty acids [59] into account by including total saturated fatty acids as a predictor, LA levels did not negatively affect DHA levels, although EPA was negatively affected. In agreement with previous observations [60] and the assumed importance of ALA for endogenous DHA synthesis, DPA-n6 was the fatty acid most strongly negatively affected by DHA intake and the ALA level. With the applied model, 84% and 90% of the variance of EPA concentrations were explained pre-intervention and post-intervention, respectively. The similarity of the coefficients of determination pre- and post-intervention suggests the absence of a significant association between DHA intake and EPA levels and does not support a quantitatively relevant retroconversion of DHA to EPA [61]. For some LC-PUFA, significant negative interactions between precursor level and genotype were seen, with the strongest effects for EPA. This could reflect that at higher ALA levels, delta 5 desaturation encoded by FADS (rs174548) could become limiting, and the precursor ALA and the conversion products compete for incorporation into the phospholipids. 

The GPL DHA percentage increased linearly with DHA intakes by 0.4% per 1 mg/kg&day from 2.4% to 5%. Without accountable DHA intake, the average DHA-% was 2.4%, which would correspond to an endogenous synthesis of 6 mg DHA per kg&day (6 times 0.4%), if extrapolation from the intake derived estimate would be valid. However, it has been found in elderly adults that DHA turnover decreases with lower intake [62], so it can be assumed that a DHA production of 6 mg/kg&day from ALA overestimates the real production. Nevertheless, the quantitative importance of ALA desaturation and elongation is obvious from the large portion of the variation in basal DHA that can be explained by the ALA levels and the FADS genotype. Considering daily ALA intakes of about 20 to 40 mg/kg in children and adolescents [63,64] and conversion of a dietary ALA to DHA up to 9% in humans [65] and even higher in mice [66], it is conceivable that in the PKU patients, a diet high in ALA can establish significant DHA levels in situations with low requirement due to low turnover. As the expression of desaturases is decreased by higher DHA levels [48], a higher DHA intake may induce lower conversion rates, explaining that in tracer studies, very low conversion rates have often been found [66].

The situation in the PKU patients without relevant DHA intake provided a unique opportunity to study LC-PUFA metabolism and the influence of DHA on cognitive function, and we obtained highly significant results, although less than the planned number of subjects could be studied, but there are some limitations. A known major determinant of cognitive function in PKU patients is the Phe level, which could not be strictly controlled during the study and may influence the test results [67]. However, statistically controlling for the Phe levels did not change the findings, so we assume that this did not obscure any association between DHA and the cognitive test results. Additional variance was introduced due to the multicentric design of the study, which was associated with slight differences of the test procedures between the centres. This precluded comparisons between the study centres but should not have affected the examination of the change over time, which was adjusted for pre study values. It might be criticized that the variance of essential fatty acid levels was mainly caused by differences between study centres, thus further factors, e.g., diets of the PKU patients, could have been different between the centres, which would require inclusion of the study centre as a confounder into the analysis. As this would no longer have allowed a meaningful examination of the associations between the fatty acids and the effects of the dietary differences were considered via the GPL fatty acid concentrations as biomarkers for fatty acid intake, this did not seem mandatory. With the availability of valid dietary intake data for the essential fatty acids, it would have been possible to establish a quantitative relationship between ALA intake and ALA plasma levels, LA intake and LA plasma levels, and how these intakes influence DHA levels. With the available information, we can describe the relationship between DHA intake and DHA levels, but we cannot compare the relative importance of ALA and DHA intake for the DHA levels.

## 5. Conclusions

In conclusion, our results indicate that DHA intakes of up to 7 mg/kg&day in PKU children lead to a linear increase of the DHA status, but not to any significant improvement of neurological or cognitive functions. Neither pre- nor post-supplementation GPL DHA was significantly associated with the tested neurological or cognitive functions. It is possible that larger dosages of DHA and higher plasma and tissue levels might be required for significant improvements. In the absence of dietary DHA, ALA supply and desaturase genotype have a significant influence on EPA and DHA levels. Although endogenous synthesis might contribute low but biologically relevant DHA levels, DHA intake should be at least 250 mg/day to obtain adequate levels in children.

## Figures and Tables

**Table 1:** Baseline description of the evaluated study participants in the individual study centres, data are given as mean ± SD or percentage.

	Munich	Birmingham	Santander	Milan	Heidelberg	Istanbul
*n*	14	22	12	9	16	9
age (years)	8.0 ± 2.4	9.2 ± 2.4	8.9 ± 2.9	10.6 ± 3.3	8.5 ± 2.1	9.3 ± 2.7
male (%)	71	59	33	67	44	67
mat. ed. ^&^ (%) ^#^	14	36	8	11	40	0
weight (kg)	26.8 ± 8.2	35.2 ± 14.8	34.3 ± 15.8	34.9 ± 14.8	32.6 ± 10.8	31.6 ± 11.4
VEP15 (ms) *	119.0 ± 13.5	108.5 ± 7.3	110.1 ± 5.5	110.5 ± 6.5	110.6 ± 5.8	115.3 ± 4.1
RPM score *	111.4 ± 20.3	101.0 ± 25.1	97.1 ± 22.0	123.3 ± 19.4	97.2 ± 22.0	85.6 ± 16.7
LOS T-score *	55.8 ± 7.5	49.3 ± 9.0	62.2 ± 10.1	58.8 ± 7.9	60.8 ± 9.0	57.7 ± 8.0
CBCL score	54.1 ± 12.2	48.6 ± 11.9	55.2 ± 9.1	51.2 ± 10.8	53.7 ± 9.9	47.1 ± 6.6
Phe (µmol/L)	230 ± 175	347 ± 201	303 ± 115	338 ± 268	323 ± 268	364 ± 113
TG (mmol/L)	1.29 ± 0.63	1.14 ± 0.68	0.77 ± 0.26	0.90 ± 0.30	1.17 ± 0.45	1.11 ± 0.41
CHOL (mmol/L)	3.52 ± 0.48	3.47 ± 0.78	3.63 ± 0.57	3.70 ± 0.52	3.78 ± 0.55	3.40 ± 0.50
DHA * (% wt/wt)	2.36 ± 0.59	2.52 ± 0.77	1.46 ± 0.42	1.85 ± 0.34	2.87 ± 1.01	1.36 ± 0.61
ARA (% wt/wt)	10.17 ± 2.00	10.04 ± 2.36	9.75 ± 2.39	10.30 ± 0.91	9.57 ± 1.68	11.48 ± 2.53
ALA * (% wt/wt)	0.35 ± 0.15	0.42 ± 0.23	0.15 ± 0.06	0.16 ± 0.08	0.37 ± 0.17	0.14 ± 0.10
LA * (% wt/wt)	22.62 ± 2.54	22.15 ± 3.20	21.45 ± 3.12	19.03 ± 2.21	21.37 ± 4.26	25.66 ± 3.76

^#^ significant differences between study centers according to chi^2^-test; * significant differences between study centers according to ANOVA; ^&^ percentage of mothers who completed school, qualifying for university.

**Table 2:** Association between study DHA intake and pre intervention values with the post intervention status of fatty acids and functional outcomes; outcomes were regressed on DHA intake and corresponding pre study value, R^2^ corresponds to the percentage of outcome variance explained by the regressors (DHA intake and pre value), significant associations (*p* < 0.01) are printed in bold.

	*n*	R^2^	Stand. β for DHA Intake	*p*	Stand. β for Pre Value	*p*
LOS T-score	78	40	−0.016	0.857	**0.644**	4.0 × 10^−10^
RPM-score	80	64	−0.108	0.118	**0.808**	5.0 × 10^−19^
VEP15 (ms)	74	57	−0.027	0.727	**0.764**	8.2 × 10^−15^
DHA-%	82	66	**0.653**	7.5 × 10^−16^	**0.532**	3.4 × 10^−12^
DHA (mg/L)	82	63	**0.614**	7.4 × 10^−14^	**0.551**	4.9 × 10^−12^
LDL/HDL	71	55	−0.055	0.495	**0.749**	7.4 × 10^−14^
Chol. (mmol/L)	76	38	0.109	0.239	**0.602**	7.5 × 10^−9^
TG (mmol/L)	79	33	0.049	0.601	**0.59**	1.3 × 10^−8^
LA (mg/L)	82	32	0.144	0.121	**0.556**	4.5 × 10^−8^
ALA (mg/L)	82	32	−0.006	0.948	**0.577**	2.4 × 10^−8^
ARA (mg/L)	82	29	−0.118	0.215	**0.526**	3.3 × 10^−7^

**Table 3:** Change of TG, Chol., LDL, HDL, VEP15, RPM-score, and LOS T-score during the intervention period, stratified into three groups according to the daily dosage of DHA (0, >0 to <1.9, ≥1.9 mg/kg&day); changes in the three groups were compared by ANOVA.

	0 (*n* ≥ 15)	>0 to <1.9 (*n* ≥ 15)	≥1.9 (*n* ≥ 42)	*p*-Value (ANOVA)
DHA (mg/L)	0.0 ± 9.1	5.1 ± 10.3	19.5 ± 13.6	1.3 × 10^−8^
TG (mmol/L)	−0.1 ± 0.7	0.0 ± 0.5	0.1 ± 0.5	0.595
Chol. (mmol/L)	−0.1 ± 0.6	0.0 ± 0.5	0.1 ± 0.5	0.795
LDL (mmol/L)	−0.3 ± 1.2	0.2 ± 0.5	−0.1 ± 0.5	0.397
HDL (mmol/L)	0.0 ± 0.3	0.0 ± 0.3	0.0 ± 0.2	0.865
VEP15 (ms)	1.3 ± 3.5	0.5 ± 8.7	−0.6 ± 4.7	0.527
RPM-score	9.5 ± 13.5	2.2 ± 15.8	1.6 ± 13.8	0.016
LOS T-score	2.9 ± 7.0	4.2 ± 6.3	0.8 ± 9.1	0.371

**Table 4:** SNPs considered in the genetic analyses and significance of their influence on pre study ARA-%, DHA-%, VEP latency, LOS T-score, RPM score, and change of DHA and VEP latency; statistically significant effects (*p* < 0.00045, considering Bonferroni adjustment are printed in bold, *n* = 82).

dbSNP	Major/Minor	Gene	HWE ^a^	MAF ^b^	%-ARA pre	DHA-% pre	VEP Latency pre	LOS pre	RPM pre	VEP Latency Change	DHA-% Change
rs174579	C/T	FADS2	0.01	0.19	**4.0 × 10^−4^**	>0.1	>0.1	>0.1	>0.1	>0.1	8.0 × 10^−3^
rs174449	A/G	FADS3	0.56	0.43	5.4 × 10^−2^	3.0 × 10^−3^	>0.1	>0.1	>0.1	>0.1	>0.1
rs968567	C/T	FADS2	0.33	0.13	2.0 × 10^−3^	>0.1	>0.1	>0.1	>0.1	>0.1	6.6 × 10^−2^
rs526126	C/G	FADS2	0.95	0.19	2.7 × 10^−2^	>0.1	>0.1	>0.1	>0.1	>0.1	>0.1
rs174455	G/A	FADS3	1.18	0.46	7.0 × 10^−2^	2.4 × 10^−2^	>0.1	>0.1	>0.1	>0.1	>0.1
rs498793	C/T	FADS2	0.71	0.35	>0.1	>0.1	>0.1	>0.1	>0.1	>0.1	>0.1
rs174570	C/T	FADS2	0.33	0.13	1.1 × 10^−2^	>0.1	>0.1	>0.1	>0.1	>0.1	>0.1
rs174561	T/C	FADS	3.04	0.28	**7.6 × 10^−9^**	3.4 × 10^−2^	>0.1	>0.1	>0.1	>0.1	9.9 × 10^−2^
rs3834458	T/del	FADS2	2.47	0.30	**1.3 × 10^−7^**	3.6 × 10^−2^	>0.1	>0.1	>0.1	>0.1	8.9 × 10^−2^
rs174548	C/G	FADS1	1.86	0.29	**7.2 × 10^−10^**	1.5 × 10^−2^	>0.1	>0.1	>0.1	>0.1	>0.1
rs174575	C/G	FADS2	0.01	0.25	**1.0 × 10^−5^**	2.5 × 10^−2^	>0.1	>0.1	>0.1	>0.1	2.0 × 10^−2^
rs174627	G/A	FADS2	0.08	0.13	>0.1	>0.1	>0.1	>0.1	>0.1	>0.1	>0.1
rs174602	T/C	FADS2	0.06	0.24	1.5 × 10^−2^	3.0 × 10^−3^	>0.1	>0.1	>0.1	>0.1	>0.1
rs174464	G/A	FADS3	0.79	0.38	4.0 × 10^−3^	7.0 × 10^−3^	>0.1	>0.1	>0.1	>0.1	>0.1
rs174468	T/C	FADS	8.37 ^c^	0.27							
rs17544159	A/C	ELOVL	0.54	0.08	>0.1	9.7 × 10^−2^	2.5 × 10^−2^	>0.1	>0.1	4.3 × 10^−2^	>0.1
rs12207094	A/T	ELOVL5	1.69	0.13	>0.1	>0.1	>0.1	>0.1	>0.1	>0.1	3.0 × 10^−2^
rs2397142	C/G	ELOVL5	0.94	0.29	>0.1	>0.1	>0.1	>0.1	>0.1	>0.1	>0.1
rs209515	G/A	ELOVL5	0.05	0.38	>0.1	>0.1	>0.1	>0.1	>0.1	>0.1	1.4 × 10^−2^
rs715441	T/C	ELOVL5	0.18	0.14	>0.1	1.9 × 10^−2^	>0.1	>0.1	>0.1	>0.1	>0.1
rs209512	T/C	ELOVL5	4.31 ^c^	0.19							
rs735860	T/C	ELOVL5	0.01	0.44	>0.1	>0.1	>0.1	>0.1	>0.1	>0.1	>0.1

^a^ chi^2^ of Hardy-Weinberg (HWE) test performed according to [25], critical value 3.86 (*p* = 0.05) ^b^ MAF: minor allele frequency ^c^ not in HWE equilibrium and not further considered.

**Table 5:** Pre and post study associations between essential fatty acid concentrations (LA, ALA), rs174548 genotype, total saturated fatty acid concentration and DHA intake (post intervention only), and major *n*-3 and *n*-6 long chain polyunsaturated fatty acid concentrations; only significant (*p* < 0.01) standardized β-coefficients are given (*n* = 82).

	R^2^	β DHA Intake	*p*-Value	β LA	*p*-Value	β ALA	*p*-Value	β rs174548	*p*-Value	β Total Saturated Fatty Acids	*p*-Value	β rs174548 × Precursor	*p*-Value
pre													
DGLA	0.69			−0.22	4.1 × 10^−3^			0.30	1.0 × 10^−6^	0.86	1.1 × 10^−18^	−0.17	4.0 × 10^−3^
ARA	0.57							−0.44	3.3 × 10^−9^	0.53	1.1 × 10^−7^		
DPA *n*-6	0.36					−0.51	2.5 × 10^−7^			0.66	7.2 × 10^−8^		
EPA	0.84			−0.33	7.6 × 10^−9^	0.83	3.5 × 10^−30^	−0.18	2.3 × 10^−5^	0.44	4.8 × 10^−12^	−0.28	7.8 × 10^−9^
DPA *n*-3	0.69			−0.21	5.6 × 10^−3^	0.61	5.2 × 10^−14^	−0.24	7.8 × 10^−5^	0.59	1.8 × 10^−9^	−0.18	5.3 × 10^−3^
DHA	0.60					0.65	9.5 × 10^−13^	−0.28	4.9 × 10^−5^	0.36	1.2 × 10^−4^	−0.23	1.4 × 10^−3^
post													
DGLA	0.61			−0.23	8.5 × 10^−3^	−0.21	3.9 × 10^−3^	0.44	3.6 × 10^−8^	0.87	1.6 × 10^−17^		
ARA	0.43							−0.39	2.7 × 10^−5^	0.52	1.0 × 10^−6^		
DPA *n*-6	0.47	−0.39	3.9 × 10^−6^			−0.52	1.7 × 10^−8^			0.68	1.9 × 10^−10^		
EPA	0.90			−0.20	4.8 × 10^−6^	0.80	1.0 × 10^−36^	−0.20	3.2 × 10^−7^	0.27	3.3 × 10^−8^	−0.18	6.7 × 10^−7^
DPA *n*-3	0.69					0.55	7.0 × 10^−13^	−0.21	1.7 × 10^−3^	0.41	2.4 × 10^−7^	−0.16	9.5 × 10^−3^
DHA	0.51	0.54	2.3 × 10^−10^			0.30	4.0 × 10^−4^			0.30	1.3 × 10^−3^		

## Supplementary Materials

The following are available online at http://www.mdpi.com/2072-6643/10/12/1944/s1, Table S1: Fatty acid composition (%) of the applied supplements with 0, 20, 43, 80, or 127 mg DHA per 500 capsule according to the analysis at the study center in Munich. Table S2: Change of blood lipids (TG, Chol.), fatty acid percentages (DHA, ARA, LA ALA), and functional outcomes (VEP latency, RPM score, LOS T-score) during the intervention period stratified according to study centers; changes in the centers were compared by ANOVA. Table S3: Latencies of visually evoked potentials (VEP) measured with pattern sizes 7.5′, 30′, 60′, and 120′ and corresponding amplitudes of the evoked potentials (mV) before (pre) and after intervention (post), all data are given as mean ± SD.
