# Supplementary material for: Determinants of Plasma Docosahexaenoic Acid Levels and Their Relationship to Neurological and Cognitive Functions in PKU Patients: A Double Blind Randomized Supplementation Study
Source: Nutrients. 2018 Dec 7;10(12):1944. doi: 10.3390/nu10121944 (PMC6316534; doi:10.3390/nu10121944)
Supplement: Supplementary file 1 [file nutrients-10-01944-s001.pdf]

Supplementary Materials:

Supplementary Table 1. Fatty acid composition (%) of the applied supplements with 0, 20, 43, 80, or 127 mg DHA per 500 capsule according to the analysis at the study center Munich

Dosage	0	20	43	80	127
C14:0	<0.1	1.0	2.2	4.1	6.4
C14:1	<0.1	<0.1	<0.1	0.1	0.1
C16:0	3.3	3.7	4.2	5.0	6.1
C16:1	0.1	0.3	0.6	0.9	1.4
C17:1	<0.1	<0.1	<0.1	<0.1	<0.1
C18:0	3.2	3.1	2.9	2.5	2.0
C18:1	85.7	80.6	74.5	65.4	53.6
C18:2n-6	5.8	5.8	5.3	4.5	3.5
C18:3n-3	0.1	0.1	0.1	0.1	0.1
C20:0	0.3	0.3	0.3	0.2	0.2
C20:1n-9	0.3	0.2	0.2	0.2	0.2
C20:3n-6	<0.1	<0.1	<0.1	<0.1	<0.1
C20:4n-6	<0.1	<0.1	<0.1	<0.1	<0.1
C20:3n-3	<0.1	<0.1	<0.1	<0.1	<0.1
C22:0	0.8	0.7	0.7	0.6	0.5
C20:5n-3	<0.1	<0.1	<0.1	<0.1	<0.1
C22:2n-6	<0.1	<0.1	<0.1	<0.1	<0.1
C22:4n-6	<0.1	<0.1	<0.1	<0.1	<0.1
C22:5n-6	<0.1	<0.1	<0.1	<0.1	<0.1
C24:1n-9	0.3	0.2	0.2	0.2	0.2
C22:5n-3	<0.1	<0.1	0.1	0.2	0.3
C22:6n-3	<0.1	3.9	8.6	15.9	25.4

Supplementary Table 2. Change of blood lipids (TG, Chol.), fatty acid percentages (DHA, ARA, LA ALA) and functional outcomes (VEP latency, RPM score, LOS T-score) during the intervention period stratified according to study centers; changes in the centers were compared by ANOVA.

	**Munich**	**Birmingham**	**Santander**	**Milan**	**Heidelberg**	**Istanbul**	**p (ANOVA)**
TG (mmol/l)	-0.22±0.49	0.20±0.57	0.18±0.35	0.12±0.39	0.07±0.74	-0.09±0.64	0.316
Chol (mmol/l)	-0.10±0.33	-0.14±0.74	-0.27±0.56	0.24±0.36	0.10±0.40	0.37±0.59	0.06
DHA (%)	1.48±1.11	0.83±1.01	0.59±1.21	1.12±1.12	1.17±0.83	1.80±1.46	0.107
ARA (%)	-0.43±1.04	-0.77±1.66	-1.29±2.16	-1.27±1.48	-0.92±1.17	-1.50±1.68	0.562
LA (%)	-1.03±2.76	-0.53±2.54	0.25±2.65	0.09±1.86	-0.61±4.42	-0.92±4.06	0.903
ALA (%)	0.07±0.19	0.05±0.22	0.00±0.06	0.01±0.07	0.05±0.20	0.01±0.11	0.875
VEP15 latency (ms)	-0.77±8.23	0.57±4.65	2.22±4.40	0.51±7.79	1.63±4.05	-3.85±4.12	0.229
RPM score	3.21±13.39	2.38±19.01	1.25±15.83	-5.63±12.37	7.19±9.83	6.67±11.18	0.44
LOS T-score	8.29±6.80	-0.05±7.92	-0.91±11.67	3.25±3.37	1.38±7.71	5.38±6.57	0.03

Supplementary Table 3. Latencies of visually evoked potentials (VEP) measured with pattern sizes 7.5′, 30′, 60′, and 120′ and corresponding amplitudes of the evoked potentials (mV) before (pre) and after intervention (post), all data are given as mean±SD.

	**n**	**pre**	**post**
VEP7 (ms)	63	119.8±9.5	118.7±8.3
VEP30 (ms)	64	108.6±9.9	109.8±7.9
VEP60 (ms)	64	105.2±14.7	106.3±7.3
VEP120 (ms)	62	106.5±10.5	105.9±10.0
VEP15 amplitude (mV)	74	20.7±11.0	20.8±12.0
VEP7amplitude (mV)	63	17.9±10.7	18.2±12.1
VEP30 amplitude (mV)	64	21.1±12.5	20.2±10.5
VEP60 amplitude (mV)	62	21.2±10.2	21.0±10.3
VEP120 amplitude (mV)	60	20.7±9.7	21.0±9.9
